# Unstable, Super Critical CO_2_–Water Displacement in Fine Grained Porous Media under Geologic Carbon Sequestration Conditions

**DOI:** 10.1038/s41598-019-47437-5

**Published:** 2019-08-02

**Authors:** R. Gooya, A. Silvestri, A. Moaddel, M. P. Andersson, S. L. S. Stipp, H. O. Sørensen

**Affiliations:** 1 Haldor Topsoe A/S, Haldor Topsoe Alle, DK-2800 Kongens Lyngby, Denmark; 2 Curtin Institute for Computation, The Institute for Geoscience Research (TIGeR), School of Molecular and Life Sciences, Curtin University, PO Box U1987, Perth, WA 6845 Australia; 3 Department of Computer Science, University of Copenhagen, Universitetsparken, 5, DK-2100 Copenhagen, Denmark; 4 Department of Chemical and Biochemical Engineering, Technical University of Denmark, Sltofts Plads, DK-2800 Kongens Lyngby, Denmark; 5 Department of Physics, Technical University of Denmark, Fysikvej DK-2800 Kongens Lyngby, Denmark

**Keywords:** Geochemistry, Carbon capture and storage, Chemical engineering

## Abstract

In this study we investigated fluid displacement water with supercritical (sc) CO_2_ in chalk under conditions close to those used for geologic CO_2_ sequestration (GCS), to answer two main questions: How much volume is available for scCO_2_ injection? And what is the main mechanism of displacement over a range of temperatures? Characterization of immiscible scCO_2_ displacement, at the pore scale in the complex microstructure in chalk reservoirs, offers a pathway to better understand the macroscopic processes at the continuum scale. Fluid behavior was simulated by solving the Navier-Stokes equations, using finite-volume methods within a pore network. The pore network was extracted from a high resolution 3D image of chalk, obtained using X-ray nanotomography. Viscous fingering dominates scCO_2_ infiltration and pores remain only partially saturated. The unstable front, developed with high capillary number, causes filling of pores aligned with the flow direction, reaching a maximum of 70% scCO_2_ saturation. The saturation rate increases with temperature but the final saturation state is the same for all investigated temperatures. The higher the saturation rate, the higher the dynamic capillary pressure coefficient. A higher dynamic capillary pressure coefficient indicates that scCO_2_ needs more time to reach capillary equilibrium in the porous medium.

## Introduction

There are many cases where detailed information about fluid flow in porous media is necessary for making informed decisions about economical or technical feasibility and for assessing risk. Simulations and predictions for carbon sequestration and contaminated groundwater remediation must all consider unpredictable fluid movement. Geological storage of CO_2_ in underground reservoirs is recognised as one of the most practical methods to decrease current atmospheric CO_2_ levels, which is an essential part of the solution for reversing global climate change^1,2^. In the sequestration process, supercritical CO_2_ is injected into porous rock formations. A critical criterion is that risk of leakage after sequestration is negligible^3–11^. Supercritical CO_2_ is normally considered a nonwetting phase and it is assumed that the aquifer is generally saturated with water, the wetting phase. There are two important questions for effective CO_2_ injection: i) How much space is available in the formation? And ii) How effective is CO_2_ migration through the pore network? To answer both of these questions, one must have a clear picture of the volume and connectivity in the pore network, within the formation.

Thermodynamic conditions can vary considerably in natural systems so the state and behaviour of CO_2_ can also vary. Depending on the environment and the injection procedure, CO_2_ can form a gas, liquid or supercritical phase. The critical temperature (T) and pressure (P) above which CO_2_ behaves as a supercritical fluid are 304.25 K and 7.39 MPa. In cold, shallow sedimentary rocks of marine origin, e.g. the Alaska north slope, T is typically ∼278 K and P is ∼7 MPa^12^ so CO_2_ would be subcritical, whereas for the Halfdan field, a deep chalk reservoir in the Danish North Sea Basin, T is ∼358 K and P is ∼40 MPa^13,14^, which is clearly in the supercritical range. In the shallow, porous basaltic rocks of the Hellisheidi injection site (CarbFix) in Iceland, where T is ∼300 K and P is 4–8 MPa, CO_2_ gas is bubbled into water and injected as an aqueous solution^15^, saturated with gas, where it rapidly reacts with the basalt to form carbonate minerals.

To describe scCO_2_ displacement in porous media, we need to know about the stability of displacement, fluid flow pathways, saturation rates, the pressure of the phases and capillary pressure. Displacement processes depend on several parameters, such as fluid viscosity, density, interfacial tension between phases and heterogeneity of the porous materials^16,17^. Two phase displacement is characterized by two dimensionless properties: the capillary number, Ca, which is the ratio of viscous to capillary forces, and the viscosity ratio, M, which represents the viscosity of the invading phase relative to the initial phase^18,19^. Based on these, the displacement process can generally be categorized into three regimes: viscous fingering, capillary fingering and stable displacement. Viscous fingering occurs at high capillary numbers and low viscosity ratios; capillary fingering happens for low Ca and high M, whereas stable displacement occurs for high Ca and M^18^.

Investigation of displacement processes can be made using 3D X-ray microtomography images^20,21^, though other methods, such as confocal microscopy^22^, have also been used. Krummel *et al*.^22^ used confocal microscopy to visualize the flow of two immiscible fluids through packed, hydrophilic glass beads and showed that, as the wetting phase pressure increases, the volume of the initial phase (residual phase) in the porous material decreases. They explained this to be a result of the increase in mobility of the nonwetting phase. Recent advances in X-ray microtomography have made it possible to observe the pore scale processes experimentally^7,23,24^. A Haines jump^25^, which is a discontinuous burst in flow that occurs at low capillary number, can be observed at the pore scale, in real time, for flow in sandstone and carbonate rocks^23,24^.

Herring *et al*.^7^ used X-ray microtomography to investigate supercritical scCO_2_ drainage in a sandstone at 311 K and 8.3 MPa. Their results showed that at M = 0.03 and Ca = 10^−8.6^, the displacement mechanism is capillary fingering. Yamabe *et al*.^21^ predicted the Haines jump for the scCO_2_–water system in a granular rock model using the lattice Boltzmann (LB) method. They assumed immiscible displacement and performed simulations at 323 K and 13.8 MPa, to emulate real scCO_2_ conditions. They showed that residual phase saturation increases with decreasing Ca. Zacharoudiou *et al*.^26^ also used Lattice Boltzmann to investigate the CO_2_–water displacement at different capillary numbers. Their results showed that higher viscosity ratio would increase the maximum saturation of CO_2_. Ramstad *et al*.^27^ used a numerical method to predict the relative permeability for sandstones and showed that the nonwetting phase velocity is overestimated because of viscous instabilities in the simulations. Their results were in good agreement with experimental data.

One of the main parameters to describe fluid displacement in the medium is capillary pressure. The measurement of capillary pressure, *P*_*c*_, depends on the medium. In a capillary tube, the situation is simple and *P*_*c*_ can be determined from the contact angle and the tube radius. In a porous material, where many pores coexist, calculation of *P*_*c*_ is much more complicated. In macroscopic experiments, the capillary pressure is determined from the inlet-outlet pressure difference. However, when using the pore geometry characterised by X-ray tomography, one needs to calculate the phase average pressure over a large number of pores.

The *P*_*c*_-saturation relationship has been shown to depend on both saturation and rate of saturation, in spite of the traditional view^28–30^, where only the dependence on the saturation is included. The effect of saturation rate on the *P*_*c*_ is known as the dynamic capillary pressure effect^31^, which establishes the speed at which flow equilibrium between CO_2_-water is reached. The slope of the capillary pressure-rate of saturation relationship represents the dynamic capillary pressure coefficient. The higher the dynamic capillary pressure coefficient, the slower the equilibrium of phases is found, i.e. longer time is needed to reach equilibrium in the CO_2_–water system.

The purpose of our work was to determine the pore scale parameters that are associated with scCO_2_ injection into a water filled porous material at pressures and temperatures relevant for CO_2_ geologic sequestration. We used X-ray nanotomography to make a 3D image of the microstructure of a sample of chalk from the Danish North Sea Basin and then we used a volume of fluid (VOF) method to investigate displacement in scCO_2_–water system. We predicted the interfacial tension between the phases, using density functional theory (DFT) with the COSMO-RS implicit solvent model. From this information, we simulated the dynamic displacement for scCO_2_–water and determined other parameters, such as pressure and saturation rate at 308 < T < 338 K.

## Results and Discussion

**Figure 1:**
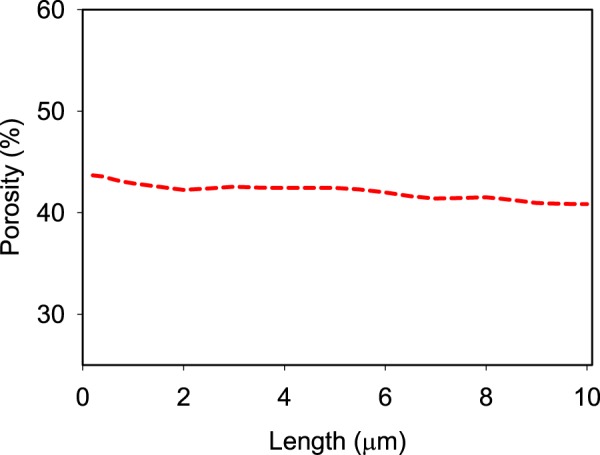
Change of porosity, determined from the selected subvolume of the 3D chalk image, as a function of the length of the subvolume in the *x* direction.

We investigated two phase flow in a sample of Hod chalk (HC #16). The 3D digital representation of the rock was obtained using X-ray holotomography at the ESRF. Data voxel size was 100 nm. We picked a subvolume within the 3D image with dimensions of 100 voxels in each direction. By subdividing the volume into smaller subvolumes, by shortening the length in the *x*-direction, we found that porosity changed by less than 3% (Fig. 1). This indicated that the sample was quite homogeneous and that the sampling volume we selected was a good representation of the material. The pore analysis of the sample^32^ indicated that the pore radii within the volume ranged between 0.2 *μ*m and 1.0 *μ*m and the average pore radius was 0.4 *μ*m. The calculated absolute permeability for the volume, determined using the method described by Gooya *et al*.^32^, was 9.06 mD, which is within the range expected for chalk^33^. Because of the sample’s demonstrated homogeneity and because of computational limitations, we chose to use a 100^3^ voxel volume, i.e. 10^3^ *μ*m^3^, for our simulations. The Ca number was from 4.0 × 10^−4^ to 1.3 × 10^−4^, M was between to 0.095 to 0.102, and a contact angle of 45°^34^ for CO_2_–water was adopted for the simulations.

**Figure 2:**
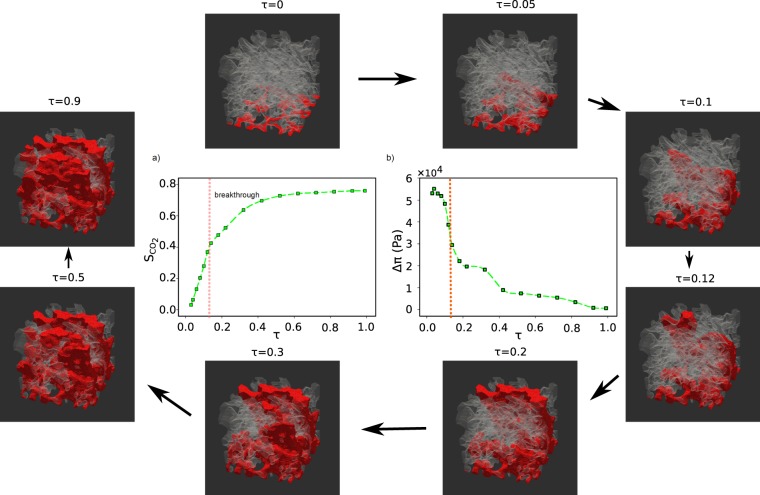
Evolution of scCO_2_–water displacement in chalk. (**a**) Saturation (S) of scCO_2_ and (**b**) pressure difference (Δ*π*) between scCO_2_ and water at the various time steps (*τ* represents the normalized time). The surrounding images are tomograms showing the pore networks as pale grey; the solid has been made to appear invisible. We see the invasion of CO_2_ (red) into the chalk as a function of time at 15 MPa and 328 K where M was 0.096 and Ca was 1.8 × 10^−4^.

Figure 2 shows the evolution in scCO_2_ saturation and the pressure difference between water and scCO_2_ during water displacement by scCO_2_. The viscosity ratio, M (the viscosity of the advancing phase, scCO_2_, divided by the viscosity of the initial phase, water), was 0.096 and Ca was 1.8 × 10^−4^. At high capillary numbers, viscous forces become dominant and the viscosity ratio determines the front stability. For M > 1, the invading fluid is more viscous than the defending fluid and the front remains stable. For M < 1, the invading fluid is the least viscous and viscous forces tend to destabilize the front. For M ≪ 1, the front is highly unstable and viscous fingers form. Weakening the viscous forces reduces the dependence on the viscosity ratio and leads to a less stable regime, i.e. more extensive fingering, in the case of M > 1, and to less evident fingers, when M < 1.

At low capillary numbers, capillary forces control the character of the displacement and pore size distribution plays an important role because it has a direct effect on the capillary forces^18,35^. In our system with high capillary number, the viscosity ratio plays an important role. When M = 0.096, scCO_2_ displaces the water with an unstable front, in the viscous fingering regime which is in agreement with the drainage displacement patterns predicted in Zacharoudiou *et al*.^26^. Figure 2 also shows that during the first time steps, one finger moves in front of the others and after breakthrough, many fingers move toward the outlet.

Figure 2b shows the pressure difference (Δ*π*) between the scCO_2_ and water averages. The average pressure is calculated by averaging the specific phase pressure over a large number of pores which is saturated by that phase. It drops suddenly after the first few time steps. The water pressure stays mostly constant during the displacement with some small jumps. The average pressure of the phases is the sum of the capillary pressure in the pores and the contribution from the viscous forces. When the capillary number is very small, the average pressure can be approximated by the capillary pressure so the difference of the average pressures represents capillary pressure under static conditions.

Another important parameter, that affects the average pressure, is the residual water that is trapped in the pores. In high capillary number regimes, where fingering dominates, all of the water cannot be displaced and there are many trapped droplets. The pressure of the trapped water is similar to the surrounding environment, which in this case, is the pressure of scCO_2_. This is because the small pressure drop across the scCO_2_–water interface is not high enough compared with the overall pressure drop across the total volume, because of viscous dissipation. Therefore, as scCO_2_ moves through the volume, the amount of trapped (residual) water increases, which causes a decrease in Δ*π*. This agrees well with previous 2D investigations by Ferrari *et al*.^35^. The small increase in the scCO_2_ average pressure at the time step, *τ* = 0.2 results to some extent from Haines jumps^25^. The Haines jump is a pore filling event that occurs when the nonwetting phase (scCO_2_) passes from a pore neck into a wider pore body, displacing the wetting phase (water).

**Figure 3:**
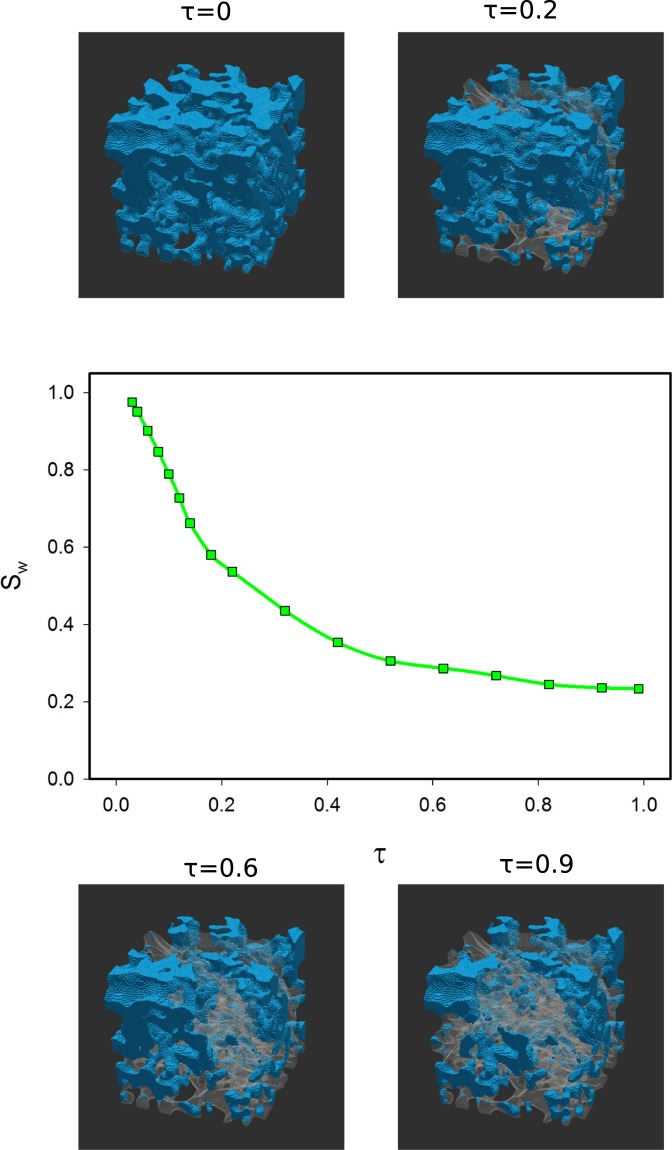
The evolution of water saturation with time at 15 MPa and 328 K where M was 0.096 and Ca was 1.8 × 10^−4^. Blue represents water; grey, scCO_2_.

**Figure 4:**
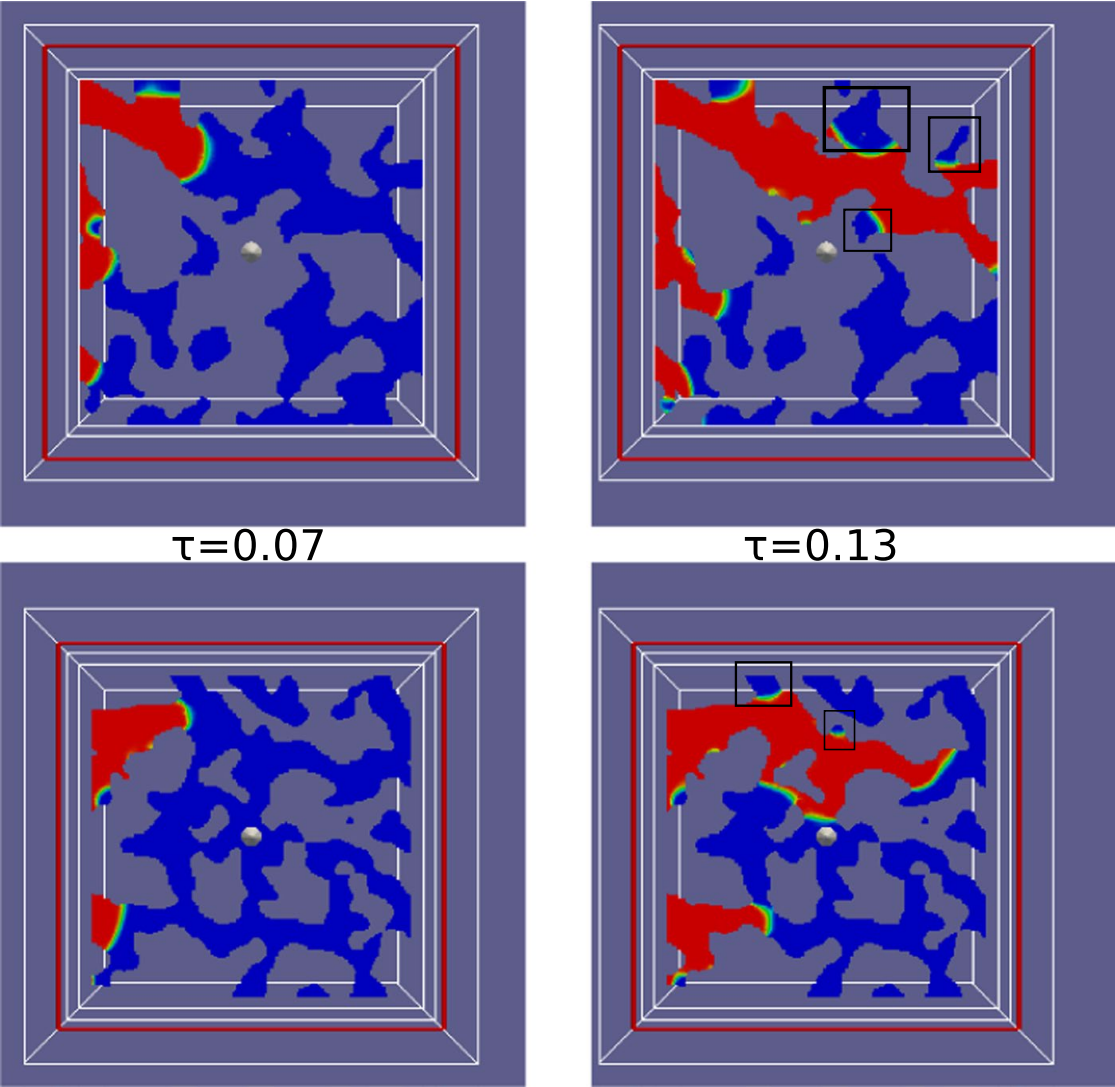
Residual water in chalk pores at 15 MPa and 328 K where M was 0.096 and Ca was 1.8 × 10^−4^. Supercritical CO_2_ displaces water from left to right. Red represents CO_2_ and blue represents water. The black squares mark examples of areas of trapped fluids. The white dot in the middle is the center of the image. There are many residual water regions in pores that are not parallel to the flow direction.

The dominance of the viscous fingering mechanism has two effects on the water residual saturation. First, most of the pores oriented along the flow direction are filled by the invading scCO_2_. Small pores are also filled because of high viscous forces. Figure 3 shows that water residual reaches 0.22 at the final time step. Second, as Fig. 4 shows, most of the residual water remains in dead end pores and pores that transect the flow direction.

**Figure 5:**
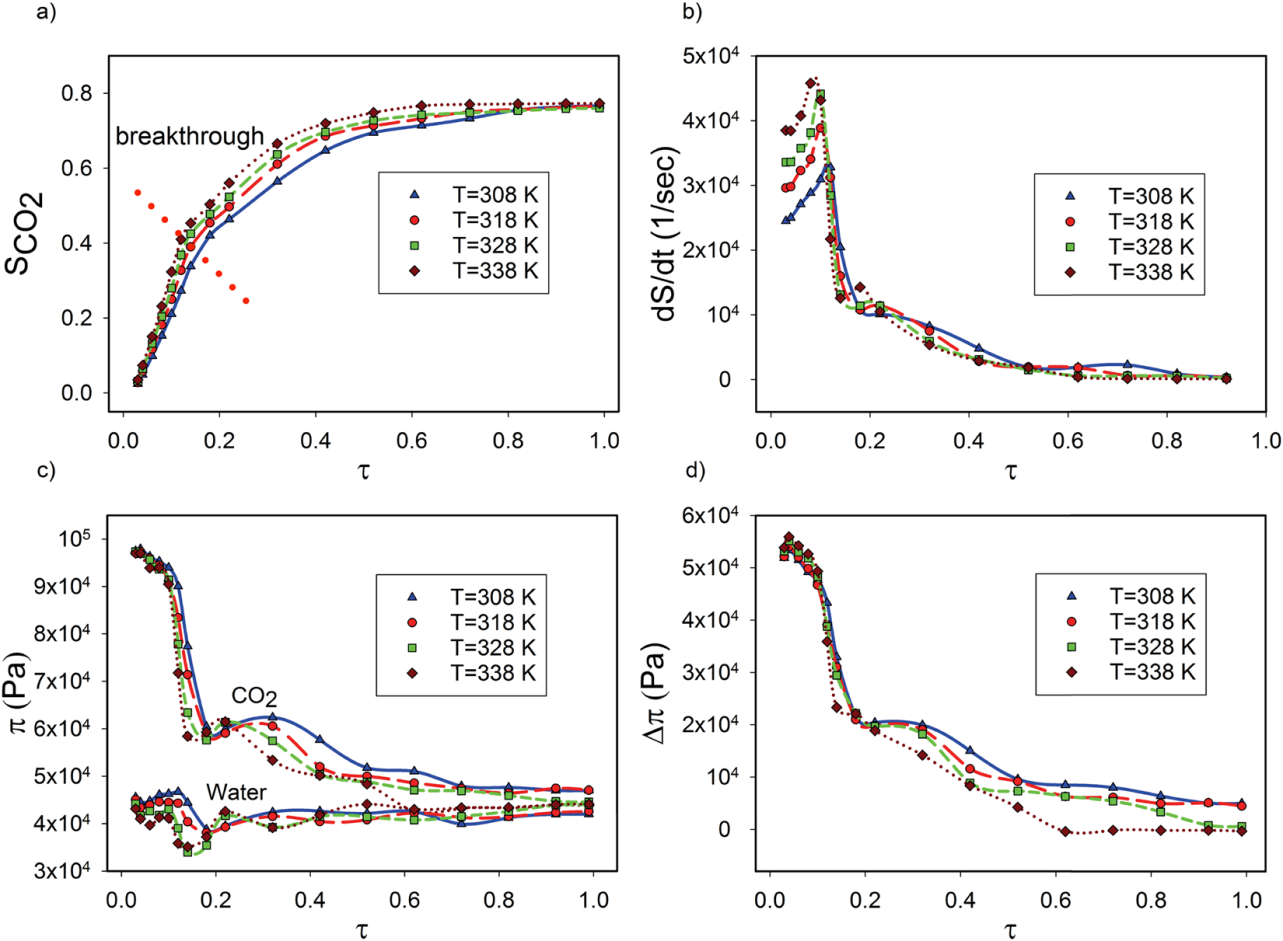
The evolution of displacement parameters with simulation time, *τ*, for four temperatures where M was 0.096 and Ca was 1.8 × 10^−4^. (**a**) Supercritical CO_2_ saturation (breakthrough occurs at the dotted red line), (**b**) saturation rate at 15 MPa, (**c**) average pressure, *π*, in the scCO_2_ and water phases and (**d**) pressure difference between scCO_2_ and water.

The evolution with time of scCO_2_ saturation for several temperatures and 15 MPa is shown in Fig. 5a, the saturation rate of scCO_2_, in Fig. 5b, water and scCO_2_ average pressure, in Fig. 5c and the pressure difference between the phases, in Fig. 5d. The scCO_2_ saturation reaches 0.77 for all temperatures (Fig. 5a). At 308 K, scCO_2_ approaches breakthrough more slowly and as temperature increases, displacement and breakthrough occur more rapidly. The saturation rate decreases after breakthrough at all temperatures as seen by Zacharoudiou *et al*.^26^. At the highest temperature we tested, T = 338 K, the system reaches maximum saturation at *τ* = 0.7. The most influential parameter for displacement is the ratio of viscosities, M, which increases as temperature rises from 308 to 318 K and then decreases (Table 1). Increasing the temperature also affects the liquid densities. The densities of scCO_2_ and water decrease by 280 kg/m^3^ and 14 kg/m^3^ over a temperature increase from 308 K to 338 K. Interfacial tension decreases gradually as temperature increases (Table 1). Higher temperature decreases the surface forces between scCO_2_ and water, thus decreasing the interfacial tension. The comparison of the capillary number at the various temperatures (Table 1) demonstrates that Ca decreases as temperature increases.

**Table 1:** Physical properties of water and CO_2_, at 15 MPa for a range of temperatures.

Temperature (K)	308	318	328	338
Water viscosity (Pa · s)	7.4 × 10^−4^	6.1 × 10^−4^	5.2 × 10^−4^	4.4 × 10^−4^
CO_2_ viscosity (Pa · s)	7.2 × 10^−5^	6.2 × 10^−5^	5.0 × 10^−5^	4.2 × 10^−5^
Water density (kg/m^3^)	994	990	986	980
CO_2_ density (kg/m^3^)	815	735	645	535
Interfacial tension (mN/m)	36.0	34.5	33.4	32.7
log(Ca)	−3.40	−3.53	−3.73	−3.87
log(M)	−1.01	−0.99	−1.01	−1.02

Figure 5b shows that the saturation rate increases with time, from *τ* = 0 to *τ* = 0.14, because of the high pressure difference between the two phases, which pushes scCO_2_ into the chalk. The increase in saturation rate ends with the first breakthrough at *τ* ∼ 0.14. Then it very rapidly decreases until *τ* = 0.2. After another small increase, it finally decreases gradually towards *τ* = 1. The saturation rate decreases gradually because the pressure difference between the phases is low.

Comparing the average pressure of scCO_2_ and water (Fig. 5c) at different temperatures shows that pressure is higher at lower temperature. The viscous dissipation occurs faster at higher temperature because scCO_2_ moves with higher velocity toward the outlet. The sharp decrease in the average pressure difference for scCO_2_ and water results mostly from the viscous dissipation and trapped water in the pore volume (Fig. 5d). The pressure difference is higher at lower temperature because of slower dissipation and less residual water in the total pore volume.

Dynamic effects on capillary pressure strongly depend on the rate of saturation (time derivative of saturation), $\frac{{\rm{d}}S}{{\rm{d}}t}$. This dependence increases for fast displacement at high capillary number. The macroscopic flow equations, known as the Darcy scale, and the experimental results describe the two phase flow properties at equilibrium conditions, where saturation no longer changes. Recent evidence shows that assuming equilibrium is probably not correct because of the dependence of parameters such as pressure on the rate of saturation^36,37^. Furthermore, saturation is determined using macroscopic data, which averages over the whole core, where there is no access to all points in the volume. Different averaging methods, such as a simple mean, simple phase average and centroid corrected average, produce different results^30^. At the pore scale, using 3D images, saturation can be determined at each point because the pore geometry is accessible and parameters can be determined for the entire volume. The higher the saturation rate, the higher the dynamic coefficient. A higher dynamic coefficient indicates that the time for reaching capillary equilibrium in the scCO_2_–water system is higher at higher temperature, which agrees with core scale studies of the dynamic capillary pressure in rocks^38^.

## Conclusions

Chalk reservoirs in the North Sea Basin have been considered for CO_2_ sequestration and the work presented here contributes to a fundamental understanding of the likely pore scale behavior of injected scCO_2_ in such very fine grained rocks. Such fine grained sediments are also found in many other geological settings. The results of the simulations show that viscous fingering dominates water displacement at all temperatures. When viscous fingering dominates, pore fluid displacement is not efficient; the available pore volume is only partially filled by CO_2_. Our modelling predicts that scCO_2_ fills the small and large pores along the flow direction and traps the initial water phase in dead end pores and in pores that are not aligned with the flow direction. Increasing temperature increases saturation rate but the final saturation state is the same for all temperatures. Faster displacement results from decreased pressure difference between scCO_2_ and water at higher temperature. When capillary equilibrium is established, CO_2_ is safely trapped in the geologic medium but at higher temperatures, the dynamic capillary coefficient is higher, which increases the time required for reaching capillary equilibrium. Lower temperatures are therefore an advantage for geologic carbon sequestration projects.

## Methods

### Tomography and image segmentation

The sample under investigation was Hod chalk (Sample HC#16) taken from a drill core from the Danish North Sea Basin. Details about the sample and the imaging conditions have been presented previously by Müter *et al*.^39^. The reconstructed image was segmented using the method described by Müter *et al*.^40^. We selected a subvolume that was 100 voxels in each dimension from the 3D image, where the voxel size was 100 nm, to serve as our model pore network for the fluid flow simulations.

### Numerical methods

The transient numerical simulation of the capillary flow was made for laminar, incompressible, immiscible, Newtonian fluids. The assumption about immiscibility is reasonable, considering that the simulations are for a short time, covering only the early phase of scCO_2_ injection and some time is required for water-scCO_2_ equilibration. The numerical method solves the Navier-Stokes equation and the volume of fluid method (VOF)^41–43^ was used to track the interface. A detailed description of the simulations is presented in the Supporting Information.

The pore volume is filled either by the nonwetting phase, *S*_*nw*_, or by the wetting phase, *S*_*w*_, where ${S}_{w}=1-{S}_{nw}$. The average pressure in the nonwetting phase is defined as *π*_*nw*_ and *π*_*w*_ for the wetting phase.

The dimensionless time, *τ*, is defined as:

1 $$\tau =\frac{{t}_{n}}{{t}_{total}},$$

where *t*_*n*_ is the current time and *t*_*total*_ is the total time of the full simulation. The saturation rate is defined as:

2 $$\frac{{\rm{d}}S}{{\rm{d}}t}{|}_{{t}_{n}}=\frac{S{|}_{n+1}-S{|}_{n}}{{t}_{n+1}-{t}_{n}}\mathrm{.}$$

The no slip boundary condition was applied at the walls and the wall boundary condition for the volume fraction function was set to zero gradient. The inlet velocity was specified such that the capillary number became ∼2 × 10^−4^. The first 0.5 *μ*m of the domain from the inlet was saturated with scCO_2_ (volume fraction is 1), leading to an overall initial scCO_2_ saturation of 0.007.

The domain was discretized as an unstructured mesh. More than twice the number of voxels were used for the mesh elements in the domain. Further mesh refinement beyond this did not improve the results. Hence, 1.2 × 10^6^ elements were used for discretizing the pore volume.

### Flow properties

The physical properties of water and scCO_2_ are important characteristics for simulating real sequestration phenomena. The simulations were performed using four temperatures above the critical temperature: 308, 318, 328 and 338 K. The pressure was kept constant at 15 MPa.

The density of supercritical CO_2_ was obtained by interpolating the experimental results of Chiquet *et al*.^44^. For density of water, we used the thermodynamic equations of Hanspal *et al*.^45^. The properties are summarized in Table 1. The viscosity of carbon dioxide was obtained using experimental data from Fenghour *et al*.^46^ and for water, which is dependent on the temperature, viscosity was calculated using the following equation:

3 $$\mu =\exp (-24.71+\frac{4209}{T}+0.04527T-3.376\times {10}^{-5}{T}^{2}),$$

where *μ* represents liquid water viscosity. The results are summarized in Table 1.

We predicted the interfacial tension between scCO_2_ and water using a recently developed quantum chemical method^47,48^ based on density functional theory and the COSMO-RS implicit solvent model. In this method, a virtual surface between scCO_2_ and water is created and the solvation contribution to the change in free energy for transferring a molecule, from the bulk phase to this interface, is calculated. For each position and orientation, the difference in free energy compared with the bulk phase is calculated as well as the corresponding interfacial area taken up by the molecule. By combining the model with an equation of state, we were able to explore pressure conditions far from atmospheric conditions. More details can been found in Silvestri *et al*.^49^.

The datasets analysed during the current study are available from the corresponding author on reasonable request.

## Supplementary information

Supplementary File
