# Supplementary material for: Unstable, Super Critical CO2–Water Displacement in Fine Grained Porous Media under Geologic Carbon Sequestration Conditions
Source: Sci Rep. 2019 Aug 2;9:11272. doi: 10.1038/s41598-019-47437-5 (PMC6677758; doi:10.1038/s41598-019-47437-5)
Supplement: Supplementary file 1 — Supplementary File [file 41598_2019_47437_MOESM1_ESM.pdf]

Supporting Information for Unstable, Super Critical CO_2–Water Displacement in Fine Grained Porous Media under Geologic Carbon Sequestration Conditions

R. Gooya[1,*], A. Silvestri[2], A. Moaddel[3], M.P. Andersson[4], S.L.S. Stipp[5], and H.O. Sørensen[5]

[1]Haldor Topsoe A/S, Haldor Topsoe Alle, DK-2800 Kongens Lyngby, Denmark
[2]Curtin Institute for Computation, The Institute for Geoscience Research (TIGeR), School of Molecular and Life Sciences, Curtin University, PO Box U1987, Perth, WA 6845, Australia
[3]Department of Computer Science, University of Copenhagen, Universitetsparken 5, DK-2100 Copenhagen Ø, Denmark
[4]Department of Chemical and Biochemical Engineering, Technical University of Denmark, Søltofts Plads, DK-2800 Kongens Lyngby, Denmark
[5]Department of Physics, Technical University of Denmark, Fysikvej, DK-2800 Kongens Lyngby, Denmark
[*]rego@topsoe.com

ABSTRACT

Numerical method

The numerical method solves the Navier-Stokes equation:

$$\frac{\partial \rho \boldsymbol{u}}{\partial t} + \bigtriangledown \cdot (\rho \boldsymbol{u}\boldsymbol{u}) = -\bigtriangledown \pi + \bigtriangledown \cdot \varepsilon + f_{SF}, \tag{1}$$

which is derived from Newton's second law, and the continuity equation, which describes the conservation of mass:

$$\bigtriangledown \cdot \boldsymbol{u} = 0. \tag{2}$$

In Eqs 1 and 2, SF represents the surface, ρ, the fluid density, $\boldsymbol{u}$, fluid velocity, t, time, ε, the stress tensor i.e.: $\mu(\bigtriangledown \boldsymbol{u} + \bigtriangledown \boldsymbol{u}^T)$, π, the pressure and f_{SF}, the surface forces.

The volume of fluid method[1–3] has been used to describe the interface. The volume describes an interface when the volume thickness get close to zero. It can be used to simulate several multiphase phenomena: the jet breakup, splashing and displacement in porous materials[2–5]. The three main aspects of the model are: i) interfacial tension, to derive surface forces, ii) advection of the interface and iii) connection to the Navier-Stokes equations. Applying a continuum surface force (CSF) model,[4] that calculates the surface force on the interface, is a common approach in the VOF method:

$$\int_{SF} \sigma k \mathbf{n} \delta \mathbf{x} dSF = \sigma k \bigtriangledown \alpha, \tag{3}$$

where σ represents interfacial tension, $\boldsymbol{n}$, the unit vector normal to the surface, δ, the delta function, $\mathbf{x}$, the position vector and k, the curvature of the surface that is determined using the local gradients of the surface normal to the interface[6]:

$$k = \bigtriangledown \cdot \left(\frac{\bigtriangledown \alpha}{|\bigtriangledown \alpha|} \right). \tag{4}$$

The CSF model acts on the volume of the interface where $0 < \alpha < 1$ and this volume force tends to the surface force when the thickness of interface tends toward zero.

The interface between two phases is advected by:

$$\frac{\partial \alpha}{\partial t} + (u \cdot \bigtriangledown)\alpha = 0, \tag{5}$$

which is solved explicitly using the velocity of the latest time step. In the VOF method, the contact angle is is taken into account in a natural way by applying the Young's equation on the triple contact point. In the triple point, Young's law is assigned in which:

$$cos\theta = \frac{\sigma_{nws} - \sigma_{ws}}{\sigma} \tag{6}$$

where σ represents the surface tension of fluid-fluid interface, σ_{nws} the surface tension of non wetting fluid-solid interface and σ_{ws} the surface tension of wetting fluid-solid interface. By applying

$$n = n_s cos\theta + t_s sin\theta \tag{7}$$

at the tripple boundary contition, the Young's law for the triple point is applied. n the normal to the fluid–fluid interface at the solid surface, n_s, the unit normal points into the solid and t_s the unit tangent to solid points into the wetting phase.

The saturation of each phase, i.e. the wetting phase, w, and the nonwetting phase, nw, in the domain is defined as:

$$S_w = \frac{1}{V}\int_V \alpha \mathrm{d}v \tag{8}$$

and

$$S_{nw} = \frac{1}{V}\int_V (1-\alpha)\mathrm{d}v, \tag{9}$$

where V refers to the pore volume. This also means that $S_w = 1 - S_{nw}$. By applying the same procedure, we can calculate the average pressures in the domain:

$$\pi_w = \frac{\int_V \pi\alpha \mathrm{d}v}{\int_V \alpha \mathrm{d}v} \tag{10}$$

and

$$\pi_{nw} = \frac{\int_V \pi(1-\alpha)\mathrm{d}v}{\int_V (1-\alpha)\mathrm{d}v} \tag{11}$$

and based on the average pressures, the pressure difference is:

$$\Delta\pi = \pi_{nw} - \pi_w \tag{12}$$

The no slip boundary condition was applied at the walls and the wall boundary condition for the volume fraction function was set to zero gradient. The inlet velocity was specified where capillary number was $\sim 2\times10^{-4}$. The first 0.5 μm of the domain in the flow direction was saturated with $scCO_2$, initial saturation = 0.007 and the inlet value for the volume fraction was set to 1, i.e. pure $scCO_2$.

The domain was discretized into unstructured mesh and more than twice the number of voxels were used for the mesh elements in the domain. Mesh refinement for getting finer mesh did not affect the results and 1.2×10^6 elements were used for discretizing the pore volume. The PISO (pressure implicit with splitting of operator)[7] algorithm was implemented in OpenFOAM libraries[8] to couple velocity and pressure. The numerical simulation procedure was the following: initialise all variables, calculate the Courant number (the ratio of flow movement to the length interval). The maximum allowed value of the Courant number was set to 0.5 (second order scheme) to ensure numerical stability for the simulations by moving the front by maximum half a cell for each time step. Then choosing the time step based on the Courant number, solve the equations with the initial values, use new values to calculate the updated properties such as viscosity and density according to the volume fraction of cells, apply new data to the equations and continue with the PISO algorithm until the final time step. The initial time step was set to 1×10^{-6} seconds. A convergence criterion of 1×10^{-6} was defined to control the iterative methods. The injection of CO_2 into 10^3 μm^3 chalk sample was simulated for 100 μs.

References

1. Hirt, C. & Nichols, B. Volume of fluid (VOF) method for the dynamics of free boundaries. *J. Comput. Phys.* **39**, 201 – 225 (1981).

2. Lafaurie, B., Nardone, C., Scardovelli, R., Zaleski, S. & Zanetti, G. Modelling merging and fragmentation in multiphase flows with {SURFER}. *J. Comput. Phys.* **113**, 134 – 147 (1994).

3. Scardovelli, R. & Zaleski, S. Direct numerical simulation of free-surface and interfacial flow. *Annu. Rev. Fluid Mech.* **31**, 567–603 (1999).

4. Brackbill, J., Kothe, D. & Zemach, C. A continuum method for modeling surface tension. *J. Comput. Phys.* **100**, 335 – 354 (1992).

5. Ferrari, A. & Lunati, I. Direct numerical simulations of interface dynamics to link capillary pressure and total surface energy. *Adv. Water Resour.* **57**, 19 – 31 (2013).

6. Warner, M. Numerical modeling of multiphase flows in microfluidics and micro process engineering: a review of methods and applications. *Microfluid. Nanofluidics* **12**, 841–886 (2012).

7. Barton, I. E. Comparison of SIMPLE- and PISO-type algorithms for transient flows. *Int. J. for Numer. Methods Fluids* **26**, 459–483 (1998).

8. OpenCFD. Opencfd ltd at esi group. *www.openfoam.com* (2013).
